# Anxiety and depression and health-related quality of life among adults with migraine: a National Population-Based Study

**DOI:** 10.3389/fpubh.2023.1241800

**Published:** 2023-10-04

**Authors:** Monira Alwhaibi, Bander Balkhi, Yazed AlRuthia

**Affiliations:** ^1^Department of Clinical Pharmacy, College of Pharmacy, King Saud University, Riyadh, Saudi Arabia; ^2^Medication Safety Research Chair, College of Pharmacy, King Saud University, Riyadh, Saudi Arabia; ^3^Pharmacoeconomics Research Unit, College of Pharmacy, King Saud University, Riyadh, Saudi Arabia

**Keywords:** anxiety, migraine, depression, quality of life, SF-12

## Abstract

**Background:**

Adults who suffer from migraines are highly susceptible to mental illnesses that may have significant association with their HRQoL. Therefore, this study aimed to investigate how anxiety and depression related to HRQoL in adults with Migraine.

**Methods:**

Data from the Medical Expenditure Panel Survey for 2017 to 2020 were used to identify adult patients 18 years of age and older with a migraine diagnosis. The Physical and Mental Component Summary (PCS & MCS) scores from the SF-12 were used to calculate HRQoL. To adjust for a wide range of variables, multivariate linear regressions were used to evaluate the association between depression and anxiety and HRQoL among adults with migraine.

**Results:**

Among the 1,713 identified adults with migraines, 11.2% experienced depression, 14.6% experienced anxiety, and 13.7% had both conditions. Compared to migraineurs who had only migraine, adults with comorbid depression and anxiety had the lowest mean scores on the PCS and MCS. Additionally, migraineurs who had depression had significantly lower HRQoL MCS scores (depression: β = −7.552, *p* < 0.001), and those with anxiety had significantly lower HRQoL MCS scores (anxiety: β = −4.844, p < 0.001) compared to those without these comorbidities. Notably, individuals with migraines who exercise had higher scores on both PCS and MCS than those who did not exercise.

**Conclusion:**

This nationally representative sample provides insights into the associations between depression and anxiety with poor HRQoL among individuals with migraines. Additionally, it revealed the negative impact of concurrent chronic diseases, and poor socioeconomic status on HRQoL, while emphasizing the beneficial effects of regular exercise. This study highlights the clinical, policy, and public health implications for improving healthcare planning, resource allocation, and promoting lifestyle changes to reduce depression and anxiety in migraine sufferers.

## 1. Introduction

Migraine is one of the most prevalent medical illnesses worldwide and has been acknowledged as having significant consequences for public health (1). It affects a substantial number of the global population, with an estimated prevalence of 15% worldwide. Migraine accounts for 4.9% of the global population illness, measured by years lived with disability (YLDs) (2). In the United States, migraine is among the top three most disabling neurological conditions, which aligns with the most disabling neurological conditions worldwide (3). Migraine is a debilitating condition that negatively impacts health, overall well-being, family relationships, work and school activities, along with a significant financial burden (4). Moreover, it is often associated with a significant impact on individuals’ psychological well-being and health-related quality of life. A substantial bidirectional link between migraine and psychiatric problems has been consistently demonstrated through research (5). Adults with migraines frequently experience anxiety and depression; they are two to four times more likely to experience depression and anxiety compared to adults in the general population (6). Depression and anxiety may increase the probability of adverse clinical outcomes, including suicidal behavior as well as treatment resistance (7). Importantly, persons at risk for suicide have been shown to have greater mean concentrations of inflammatory mediators in both the brain and the periphery when compared to non-suicidal participants (7). In addition, coexisting depression and anxiety in adults with migraine add to their financial burden, increase healthcare utilization, increase migraine illness burden, psychosocial dysfunction, and may impair migraine sufferers’ health-related quality of life (7–16).

Health-related quality of life (HRQoL) is a crucial outcome factor for migraine sufferers. It encompasses various aspects of an individual’s physical, mental, and social well-being that are influenced by their health status (17). HRQoL has been defined as “how well a person functions in their life and their perceived wellbeing in physical, mental, and social domains of health (18)”. The multidimensional nature of HRQoL makes it an essential outcome measure for assessing the overall impact of chronic conditions such as migraine. One of the main goals in adults with migraine may be to maintain a good health-related quality of life despite the challenges brought on by migraines. Therefore, determining the causes of a poorer HRQoL is crucial, especially considering mental health comorbidities. Few studies evaluated how migraine patients’ HRQoL. Population-based studies in the United States (US) and the United Kingdom (UK), with similar designs showed that those with depression and migraines scored worse on both the physical and mental components of HRQoL than people without depression (8). Evidence from a nationwide Canadian Community Health Survey of participants with migraine demonstrated that health-related outcomes were worse in those with migraines and a psychiatric disease than in individuals with either condition alone (10, 16). In a sample of 1,957 migraineurs, Lanteri-Minet et al. found that concurrent anxiety in patients with depression resulted in a considerably lower HRQoL (13).

Limited research have examined how migraine patients’ HRQoL is related to depression and/or anxiety (8, 10, 13, 16). Even though these studies added to the body of evidence regarding the effect of depression and/or anxiety on HRQoL in adults with migraine, our investigation included national US data for individuals with migraine. In addition, earlier research studies used self-reported scales to assess anxiety and depression, whereas this study employed clinical diagnostic codes. Furthermore, we have considered various confounders that could influence the relationship between depression, anxiety, and HRQoL, including socioeconomic variables, access to care, physical activity, and medical comorbidities. We hypothesized that anxiety and depression are associated with poor HRQoL among adults with migraine. Understanding the intricate relationship between migraine, depression, anxiety, and HRQoL is crucial for healthcare providers and researchers to optimize patient care and develop effective interventions that address both the physical and psychological aspects of their condition.

## 2. Methods

### 2.1. Study design and data

A cross-sectional analysis was carried out using information from the Medical Expenditure Panel Survey (MEPS) for the years 2017, 2018, 2019, and 2020. MEPS is a national survey that collects data from the non-institutionalized US civilian population on sociodemographic traits, health insurance, medical problems, medication use, and other health services.

### 2.2. Study population

The study inclusion criteria include: (1) adults between the ages of 18 and 64, (2) diagnosed with migraine, (3) were alive during the calendar years, and (4) had no missing data for HRQoL. We have excluded individuals with no migraine diagnosis and those with missing data for HRQoL. Migraine diagnosis was identified from MEPS data using the International Classification of Diseases, Ninth Revision, Clinical Modification (ICD-9-CM) clinical diagnosis codes,

### 2.3. Measures

#### 2.3.1. Outcome: health-related quality of life (HRQoL)

Adults who took part in the MEPS and were at least 18 years old had to self-administer questionnaires to determine their HRQoL. Both the physical and mental dimensions of HRQoL were evaluated using the Physical Component Summary (PCS) and Mental Component Summary (MCS) of the questionnaire from the Short-Form 12 Version 2 (SF-12 V2) (19). Higher scores on the PCS and MCS indicate better physical and mental HRQoL, respectively.

#### 2.3.2. Variables

Adults with migraine were divided into four mutually exclusive groups (e.g., migraine only, migraine and anxiety, migraine and depression, migraine and both disorders). Other variables included sociodemographic factors (gender, age, race or ethnicity, marital status, income, region of residence, education level, health insurance, medication insurance, employment status, and poverty status), perceived physical health, concurrent chronic illnesses, and physical exercise.

### 2.4. Statistical analyses

Descriptive statistics, such as mean, standard deviation, frequencies, ad percentages, were used present the baseline characteristics of the study sample. Chi-square tests were employed to determine the variations in individual characteristics among the migraine groups. ANOVA was used to find mean variations in HRQoL by migraine groups. We controlled for all independent factors in the multivariable linear regressions to assess the relationship between migraine groups and HRQoL. Statistical significance was defined as a value of p less than 0.05. The MEPS complex survey design was taken into account for all estimations in the statistical analyses by integrating person-level weights and variance adjustment weights (strata and primary sample unit) from the MEPS. The data were analyzed with SAS 9.4 (SAS Institute Inc., Cary, NC, United States).

## 3. Results

### 3.1. Characteristics of the study sample

Table 1 lists the characteristics of the study sample, 1,713 adults with migraine; the majorities of the study sample’s participants were women (83.5%), aged 18 to 39 (36.7%), employed (71.8%), and had at higher than high school diploma (92.7%). About 11.0% of migraine affected persons also experienced depression, 14.6% had anxiety, and 13.7% had both illnesses. Compared to men, women with migraine exhibited significantly greater anxiety rates (15.2% vs. 11.4%) and comorbid depression and anxiety (14.9% vs. 7.5%, value of *p* = 0.028). Additionally, compared to employed migraine patients, those who were unemployed had considerably more significant rates of depression (15.8% vs. 9.4%) and comorbid anxiety and depression (23.7% vs. 9.8%, value of *p* <0.0001). In addition, comorbid depression and anxiety were substantially more common in persons with migraine and comorbidities (diabetes, hyperlipidemia, asthma, COPD, arthritis, GERD) than in adults without these comorbidities (value of *p* <0.0001).

**Table 1:** Characteristics of the study sample, raw percentage of characteristics by migraine group.

	Total sample		Migraine only		Migraine & Depression		Migraine & Anxiety		Migraine & Dep & Anxiety			
	*N*	Wt.%	*N*	Wt.%	*N*	Wt.%	*N*	Wt.%	*N*	Wt.%	*p* value	sig
**All**												
**Age in years**	1,713	100	995	60.5	192	11.2	245	14.6	239	13.7		
18–39	582	36.7	365	62.3	50	9.3	84	13.9	83	14.5	0.574	
40–49	470	27.9	286	60.1	51	10.5	75	16.6	58	12.9		
50–64	619	35.4	344	59.1	91	13.8	86	13.7	98	13.5		
**Gender**												
Women	1,409	83.5	820	59.0	159	10.8	217	15.2	213	14.9	0.028	*
Men	262	16.5	175	68.1	33	12.9	28	11.4	26	7.5		
**Race/ethnicity**												
White	1,118	75.0	627	57.3	131	11.6	175	15.6	185	15.6	0.006	**
African American	185	8.3	121	73.6	24	9.5	23	9.2	17	7.7		
Latino	256	10.1	179	72.5	21	7.7	33	13.4	23	6.4		
others	112	6.6	68	63.1	16	14.2	14	11.8	14	10.9		
**Marital status**												
Married	877	55.6	571	65.5	94	10.2	130	15.2	82	9.1	<0.0001	***
Wid/Sep/Div	421	23.2	205	48.7	65	14.6	60	13.5	91	23.2		
Never married	373	21.1	219	60.6	33	10.0	55	14.0	66	15.5		
**Education level**												
LT HS	47	1.7	31	66.5	5	11.9	4	9.6	7	12.0	0.005	**
HS	129	5.3	65	46.4	14	10.9	18	13.2	32	29.5		
> HS	1,487	92.7	894	61.2	173	11.2	223	14.8	197	12.8		
**Region**												
Northeast	298	18.1	187	66.2	28	7.8	32	9.4	51	16.6	0.033	*
Mid-west	403	22.6	231	59.5	64	16.2	46	10.9	62	13.4		
South	540	34.5	315	60.3	63	10.7	90	17.1	72	11.9		
West	430	24.8	262	57.7	37	9.8	77	18.2	54	14.4		
**Employment**												
Employed	1,116	71.8	743	66.0	105	9.4	161	14.8	107	9.8	<0.0001	***
Not employed	555	28.2	252	46.6	87	15.8	84	13.9	132	23.7		
**Poverty status** ^**¥**^												
Poor	289	13.2	146	47.3	44	17.5	30	7.8	69	27.4	<0.0001	***
Near Poor	285	14.4	167	58.8	25	7.1	40	14.9	53	19.2		
Middle Income	438	26.9	237	55.1	46	10.6	81	17.9	74	16.4		
High Income	659	45.6	445	68.1	77	11.0	94	14.4	43	6.4		
**Health insurance**												
Private	1,147	75.2	722	64.0	128	10.6	178	15.2	119	10.2	<0.0001	***
Public	459	21.6	227	48.5	58	12.0	60	12.5	114	27.0		
Uninsured	65	3.3	46	61.0	6	18.4	7	13.5	6	7.1		
**Rx Insurance**												
Rx insurance	994	66.2	630	64.4	113	10.9	155	15.4	96	9.2	<0.0001	***
No Rx insurance	677	33.8	365	53.0	79	11.7	90	12.8	143	22.5		
**General health**												
Excellent/very good	656	43.4	482	73.3	58	8.9	72	11.1	44	6.6	<0.0001	***
Good	538	30.6	308	56.0	64	12.0	91	17.4	75	14.6		
Fair/poor	477	26.0	205	44.4	70	13.9	82	17.0	120	24.6		
**Physical activity**												
3/week	685	42.3	448	66.0	63	9.5	89	12.8	85	11.7	0.034	*
No exercise	983	57.5	545	56.6	128	12.3	156	15.9	154	15.2		
**Heart**												
Yes	100	5.5	49	50.5	13	18.3	13	13.4	25	17.7	0.267	
No	1,571	94.5	946	61.1	179	10.8	232	14.6	214	13.5		
**Hypertension**												
Yes	389	19.9	208	56.4	53	13.5	63	16.0	65	14.1	0.511	
No	1,282	80.1	787	61.6	139	10.6	182	14.2	174	13.6		
**Diabetes**												
Yes	147	7.2	60	36.6	26	16.1	21	13.4	40	34.0	<0.0001	***
No	1,524	92.8	935	62.4	166	10.8	224	14.7	199	12.1		
**Hyperlipidemia**												
Yes	281	15.7	127	45.9	46	16.2	53	19.9	55	18.0	0.001	**
No	1,390	84.3	868	63.3	146	10.2	192	13.6	184	12.9		
**Asthma**												
Yes	309	17.7	144	47.2	41	14.4	52	15.0	72	23.3	<0.0001	***
No	1,362	82.3	851	63.4	151	10.5	193	14.5	167	11.6		
**COPD**												
Yes	140	8.0	47	33.8	17	10.4	25	18.3	51	37.5	<0.0001	***
No	1,531	92.0	948	62.9	175	11.3	220	14.2	188	11.7		
**Arthritis**												
Yes	246	13.3	101	40.5	41	17.4	41	18.2	63	23.9	<0.0001	***
No	1,425	86.7	894	63.6	151	10.2	204	14.0	176	12.2		
**GERD**												
Yes	260	15.5	114	44.0	45	18.1	57	22.1	44	15.9	<0.0001	***
No	1,411	84.5	881	63.6	147	9.9	188	13.2	195	13.3		

Asterisks (*) represent significant differences in migraine groups from chi-square tests; ***p < 0.001; **0.001 ≤ *p* < 0.01; *0.01 ≤ *p* < 0.05.

Wt, weighted; LT, less than; GT, greater than; Rx: Medication; Wid./Div./Sep.: widowed, divorced, and separated.

¥ Based on the federal poverty line (FPL): poor (100% FPL), near poor (100 to 200% FPL), middle income (200 to 400% FPL), and high income (>400% FPL).

### 3.2. Health-related quality of life and migraine groups

The PCS and MCS ratings for HRQoL significantly differed between migraine groups (Table 2). For instance, the mean PCS score was lower in migraine patients with both depression and anxiety (Mean = 36.43, SE =1.43) compared to other groups (41.52 for migraine only, 38.76 for migraine and depression, and 10.41 for migraine & anxiety). For MCS score, it was lower in migraine patients with both depression and anxiety compared to other groups (33.20 for migraine and depression and anxiety, 43.60 for migraine only, 38.35 for migraine and depression, and 39.03 for migraine & anxiety).

**Table 2:** Weighted means of health-related quality of life scores by migraine groups.

	Total Sample		Migraine Only		Migraine & Depression		Migraine & Anxiety		Migraine & Depression & Anxiety		
	Mean	SD	Mean	SE	Mean	SE	Mean	SE	Mean	SE	*p* value
HRQoL											
PCS	38.69	19.9	41.52	0.80	38.76	1.62	40.41	1.65	36.43	1.43	0.0004
MCS	39.59	19.8	43.60	0.78	38.35	1.44	39.03	1.58	33.20	1.37	0.0004

ANOVA was used to analyze mean differences by migraine groups.

HRQoL, Health-related Quality of Life; MCS, Mental Component Summary; PCS, Physical Component Summary; SE, Standard Error; SD, Standard Deviation; Sig, Significance.

### 3.3. Health-related quality of life among migraine groups from adjusted analysis

Table 3 displays the adjusted relationship between migraine groups and HRQoL. A significantly lower HRQoL was found in migraine patients with depression (MCS: β = −7.552, value of *p*<0.0001), with anxiety (MCS: β = −4.844, value of p<0.0001), with both depression and anxiety (MCS: β = −9.806, value of *p*<0.0001) compared to those with migraine only after adjusting for all independent variables.

**Table 3:** Parameter estimates from adjusted multivariate linear regressions on hrqol among adults with Migraine, MEPS 2017–2020.

	Health-related quality of life					
	PCS			MCS		
	Regression Coefficients	SE	Sig.	Regression Coefficients	SE	Sig.
**Migraine groups**						
Migraine & depression	−0.013	0.184		−7.552	0.116	***
Migraine & anxiety	1.397	0.065	***	−4.844	0.065	***
Migraine & depression & anxiety	−0.059	0.054		−9.806	0.059	***
Migraine only (Ref.)						
**Age in years**						
18–39	2.819	0.019	***	−1.209	0.044	***
40–49	2.180	0.089	***	−1.159	0.047	***
50–64 (Ref.)						
**Gender**						
Women	−1.518	0.049	***	1.274	0.033	***
Men (Ref.)						
**Race/ethnicity**						
White	1.563	0.258	***	0.257	0.262	
African American	0.650	0.227	**	−0.343	0.173	*
Latino	1.255	0.315	***	0.578	0.270	*
Others (Ref.)						
**Marital status**						
Married	−1.228	0.071	***	−0.593	0.115	***
Widow/Separate/Divorce	−0.119	0.038	**	−1.276	0.101	***
Never married (Ref.)						
**Education level**						
> HS	2.193	0.485	***	2.217	0.120	***
HS	−0.423	0.511		4.316	0.128	***
LT HS (Ref.)						
**Region**						
Northeast	−0.146	0.033	***	1.466	0.039	***
Mid-west	0.124	0.050	*	1.344	0.026	***
South	−0.220	0.067	**	0.535	0.054	***
West (Ref.)						
**Employment**						
Employed	3.180	0.072	***	−0.206	0.060	***
Not employed (Ref.)						
**Poverty status**						
Poor	−1.166	0.199	***	−1.451	0.134	***
Near Poor	−0.149	0.086		−2.004	0.146	***
Middle Income	−1.250	0.032	***	0.463	0.018	***
High Income (Ref.)						
**Health Insurance**						
Private	−0.513	0.066	***	1.091	0.082	***
Public	−2.934	0.125	***	−1.341	0.079	***
Uninsured (Ref.)						
**Rx Insurance**						
Rx insurance	−0.720	0.046	***	−0.121	0.039	**
No Rx insurance (Ref.)						
**General health**						
Excellent/very good	12.682	0.161	***	9.040	0.138	***
Good	7.400	0.128	***	6.051	0.067	***
Fair/poor (Ref.)						
**Physical activity**						
3/week	1.491	0.059	***	1.878	0.103	***
**Heart**						
Yes	−5.262	0.025	***	−0.251	0.033	***
**Hypertension**						
Yes	−2.055	0.026	***	0.300	0.022	***
**Diabetes**						
Yes	1.289	0.044	***	−0.413	0.018	***
**Hyperlipidemia**						
Yes	0.237	0.083	**	−1.642	0.030	***
**Asthma**						
Yes	−1.191	0.134	***	0.625	0.111	***
**COPD**						
Yes	0.102	0.128		0.124	0.031	***
**Arthritis**						
Yes	−4.186	0.032	***	−0.727	0.145	***
**GERD**						
Yes	−0.932	0.049	***	0.805	0.037	***

Asterisks denote statistical significance in parameter estimates from multivariate linear regressions on health-related quality of life, ***p < 0.001; **0.001 ≤ *p* < 0.01; *0.01 ≤ *p* < 0.05.

LT, less than; GT, greater than; Rx: Medication; Ref: reference group; SE, Standard Error; Sig, Significance.

Young age was positively associated with PCS and negatively associated with MCS. However, the female gender was negatively associated with PCS and positively associated with MCS. Factors positively associated with HRQoL included employment, perceived general health, and physical activity. For example, adults who exercise three times per week have a higher HRQoL in both physical health summary score (PCS: β = 1.491, value of *p*<0.0001) and mental health summary score (MCS: β = 1.878, value of p<0.0001) compared to those who did not exercise. Low income was negatively associated with both PCS and MCS. Individuals with concurrent comorbidities including heart disease, hypertension, asthma, arthritis, and GERD have a lower HRQoL in the physical health summary score than those without those comorbidities value of *p* <0.0001).

## 4. Discussion

The results presented here provide insight into how mental and physical comorbidity related to HRQoL in migraine sufferers and reinforces the negative relationship between HRQoL and comorbid depression and anxiety that has been investigated in multiple studies but with variable effect sizes (20). However, our research used a nationally representative sample from the U.S. that examined the relationship between migraine sufferers and the mental and physical components of HRQoL separately, fulfilling some gaps and uncertainties regarding the relationship between different domains of HRQoL and comorbid depression and anxiety.

The HRQoL had been evaluated for four migraine groups: migraine only, migraine and depression, migraine and anxiety, and migraine with both depression and anxiety. Comparing these groups, we found that individuals with depression and anxiety had lower HRQoL than those who just had one of these comorbid conditions, or those without any of these comorbidities. The burden on individuals’ quality of life extends beyond the physical symptoms of migraines, encompassing the psychological dimension. Specifically, anxiety was found to be related to the physical domain of the HQRoL, while those with both depression and anxiety related to the mental health domain of HRQoL in migraine sufferers. This finding suggests that anxiety and depression co-morbidity have cumulative impacts on HRQoL in persons with migraine. The results of our analysis are consistent with other previously published studies (8, 10, 13, 16). Jette et al., in a nationwide sample of 3,984 Canadian adults revealed lower HRQoL in people with psychiatric illness and migraines (10). Additionally, Lanteri-Minet et al. observed that concurrent anxiety and depression significantly reduced HRQoL in a nationally population-based survey they conducted in France (13). Lipton et al., in a population-based study with 246 migraine sufferers in the U.S., found that those with depression and migraines had lower scores on both the physical and mental aspects of HRQoL than people without depression (8).

This study adds to the existing body of evidence using data that is nationally representative of the US population. This finding emphasizes the need for comprehensive care that addresses both migraine therapy and mental health concerns to identify people who might benefit from early intervention, which often ignores. The Lancet series on global mental health concluded that mental health should be linked to all health facets (21). Therefore, the findings of our study should support the early screening, diagnosis, and management of depression and anxiety among migraineurs as this might significantly alleviate the suffering of the patients, lessen their symptoms, and enhance HRQoL. Various screening tools are available to assess general psychiatric symptoms. Screening becomes particularly important when patients or their families report symptoms of depression and anxiety, or when clinicians observe dysphoric or anxious moods (22).

A study using American Registry for Migraine Research assessed the symptoms of anxiety and depression using HRQOL questionnaire along with assessment of functional impairment at work (23). The analysis revealed that greater severity of depression symptoms was associated with increased migraine-related disability, pain interference, work impairment (including absenteeism and presenteeism), and work productivity impairment. This loss of productivity can have a substantial economic impact on individuals, employers, and the overall economy. Previous evidence demonstrated that comorbid psychiatric conditions such as depression and anxiety may contribute to the increased healthcare costs associated with migraine (9, 24). Thus, treatment strategies should not only focus on reducing the frequency and severity of migraines but also address the associated anxiety and depression symptoms.

Integrating mental health support into migraine management improves outcomes and enhances HRQoL. Recent findings suggest that a brief group education and supportive self-management program may improve HRQoL in individuals with chronic migraine (25). Moreover, social support was proven to play a significant role in the context of migraines, depression, and anxiety. It was observed that higher levels of social support were associated with lower scores of depression and anxiety in individuals with migraines. Therefore, fostering social support networks and interventions targeting social support may be beneficial in improving the overall well-being and mental health of individuals with migraines (26).

Untreated depression and anxiety can disrupt sleep, leading to a negative cycle of sleep deprivation and worsening mental health symptoms (27). Disturbances in sleep, such as insomnia or disrupted sleep patterns, are commonly observed among migraineurs, with 85.9% reporting clinically significant poor sleep quality. It is also prevalent in individuals with depression and anxiety. Lack of quality sleep can worsen symptoms of depression and anxiety and make it harder to cope with daily life. The significance of poor sleep quality in individuals with episodic migraine has been reported in the literature (27). The independent contribution of sleep quality to functional impairment emphasizes the importance of addressing sleep disturbances in managing migraine. A comprehensive management approach targeting depression, anxiety, and sleep quality may lead to improvements (27). Further research is needed to explore alternative interventions and strategies targeting sleep quality and insomnia for optimizing HRQoL in this population.

In our analysis, 80% of the adult migraine were women, which aligns with estimates from a 12-year Danish study that revealed a female-to-male ratio of 6.2:1 (28). This study found that feminine gender, unemployment, low income, and physical comorbidities were all substantially linked to poor HRQoL. Physical comorbidities are a crucial issue in the context of migraine. In the physical health summary score, people with concurrent comorbidities such as heart disease, hypertension, asthma, arthritis, and GERD scored significantly lower on the HRQoL scale than people without those comorbidities. These comorbidities are highly prevalent among adults with migraine, and related to experiencing more frequent migraine symptoms and more negative life events (29). Another important finding is the female gender and the low HRQoL in the physical health summary score and low income was related to low scores in both the physical and mental health summary. Similar findings were reported from a study using the Community Health Survey (CCHS), Brna et al. evaluated the factors influencing HRQOL among Canadians, and reported low income and female gender are related to poor HRQoL (16). The association between HRQoL and various demographic, socioeconomics and lifestyle factors further emphasizes the multidimensional nature of the impact of depression and anxiety on migraine patients (30). The associations between demographic and lifestyle factors with HRQoL outcomes in migraine patients have important implications for clinical practice. Understanding these factors can guide healthcare providers in identifying individuals who may be at higher risk of experiencing poor HRQoL and tailoring interventions accordingly. For example, targeting younger individuals with additional support and coping strategies may help mitigate the negative impact of migraines on their quality of life. Similarly, promoting physical activity and providing resources to improve financial well-being may contribute to better HRQoL outcomes in this population. The findings of this study also emphasize the need for a multidisciplinary approach to migraine management that incorporates pharmacological, psychological, and lifestyle interventions tailored to individual patient needs can help mitigate the negative impact of these comorbid conditions and enhance overall well-being.

### 4.1. Study strengths and limitations

This is the first study to assess the HRQoL between four groups of migraineurs. It evaluated how depression and/or anxiety affected the HRQoL of individuals with migraine using a credible and accurate evaluation method. This study also considered various variables, including sociodemographic traits, physical activity, health insurance coverage, and coexisting chronic illnesses. In addition, employing a nationally representative sample of adults with migraine allowed us to obtain accurate estimates of the relationship between depression and/or anxiety among adults with migraine and HRQoL. The present study has certain limitations when interpreting the study’s findings. Migraine-related variables, such as the severity of the disease, migraine treatment, pain, and fatigue symptoms can interfere with a person’s ability to function normally and may have a negative correlation with HRQoL; however, they are not present in MEPS and were not adjusted in the analysis. The study is limited by the use of a generic HRQoL tool rather than a disease-specific assessment tool, which may more accurately represent clinical state. However, the SF-12 questionnaire has been widely used to assess HRQoL worldwide.

Furthermore, MEPS does not contain data on the intensity of depression or anxiety, which could impact the HRQoL of adults with migraine; as a result, the regression analysis did not account for this information. Additionally, since this study used cross-sectional data, the associations observed do not imply causality; thus, a prospective longitudinal design is required to evaluate the causal relationship. Finally, this study only involved adults; hence the findings cannot be generalized to older adult individuals.

### 4.2. Clinical practice and public health implications

Healthcare professionals and policymakers can use the information presented in this study at the federal and state levels to enhance healthcare planning and resource allocation, including research funding, to prevent and minimize the harmful effect of depression and anxiety in individuals with migraine illness. In addition, this study suggests that routine screening for depression and anxiety is necessary for neurologists and other healthcare professionals to identify adults at a greater risk of having lower HRQoL, particularly for women and those with low poverty status and comorbidities. Healthcare professionals should thus treat depression and anxiety because early detection and therapy can improve HRQoL and other migraine health outcomes. Additionally, coordinated care among healthcare professionals and the provision of patient-centered information are crucial components of patient care, which can help adults with migraines have better HRQoL and lower levels of depression and anxiety. This study has public health implications for encouraging lifestyle changes like physical activity, managing obesity, eating a healthy diet, leading a better lifestyle (e.g., getting enough sleep), using mindfulness-based meditation techniques, and reducing the amount of human-technology interaction. These lifestyle changes can significantly lower the frequency and severity of mental health issues and migraine attacks (31, 32). For research implications, future studies with large sample sizes are needed to evaluate the relationship between migraine, depression, anxiety, and HRQoL in this population. There is a significant unmet need for additional research to determine the effectiveness of treating these mental illnesses in improving HRQoL for patients with migraine.

## 5. Conclusion

Adults with migraines may have worse HRQoL due to depression and anxiety. Underestimating the effects of these psychological distresses could harm migraine health outcomes. It is crucial to prioritize early diagnosis and treatment of depression and anxiety, especially for women, those with low-income status, and those with comorbidities. By addressing and managing anxiety and depression levels effectively, we can enhance the HRQoL of adults with migraines and promote their overall well-being. This study highlights the implications for clinical practice and policy to improve healthcare planning and resource allocation. It has important public health implications for encouraging lifestyle modifications that dramatically reduce the frequency and intensity of mental health problems and migraine attacks.

## Data availability statement

Publicly available datasets were analyzed in this study. This data can be found at: https://meps.ahrq.gov/data_stats/download_data_files.jsp.

## Ethics statement

Ethical approval was not required for the study involving humans in accordance with the local legislation and institutional requirements. Written informed consent to participate in this study was not required from the participants or the participants' legal guardians/next of kin in accordance with the national legislation and the institutional requirements.

## Author contributions

MA: developing the design, searching the literature, writing the manuscript, and analyzing the findings. BB: searching the literature, writing the paper, and analyzing the findings. The final manuscript was read and approved by each author. YA: searching the literature, writing the paper, and analyzing the findings. All authors contributed to the article and approved the submitted version.

## Abbreviations

HRQoL: Health-Related Quality of Life
MCS: mental health component score
PCS: physical health component score.
